# In vivo and in vitro validation of powdery mildew resistance in garden pea genotypes

**DOI:** 10.1038/s41598-023-28184-0

**Published:** 2023-02-08

**Authors:** Chanchal Rana, Akhilesh Sharma, Rajeev Rathour, Bansuli, Devinder Kumar Banyal, Ranbir Singh Rana, Parveen Sharma

**Affiliations:** 1 Department of Vegetable Science and Floriculture, Chaudhary Sarwan Kumar Himachal Pradesh Krishi Vishvavidyalaya, Palampur, Himachal Pradesh 176062 India; 2 Department of Agricultural Biotechnology, Chaudhary Sarwan Kumar Himachal Pradesh Krishi Vishvavidyalaya, Palampur, Himachal Pradesh 176062 India; 3 Department of Plant Pathology, Chaudhary Sarwan Kumar Himachal Pradesh Krishi Vishvavidyalaya, Palampur, Himachal Pradesh 176062 India; 4 Centre for Geo-Informatics Research and Training, Chaudhary Sarwan Kumar Himachal Pradesh Krishi Vishvavidyalaya, Palampur, Himachal Pradesh 176062 India

**Keywords:** Biotechnology, Genetics, Plant sciences

## Abstract

Powdery mildew is one of the serious diseases of garden pea which causes a large number of yield losses. Genetic resistance is quite effective, being cost-effective and environment friendly than fungicide applications. In the present studies an initial attempt has been made to identify resistant genotypes against powdery mildew disease developed from hybridization followed by validation of the disease. The experimental material comprised of 48 genotypes that includes 44 advanced breeding lines was evaluated for powdery mildew incidence in Randomized Complete Block Design with three replications at two locations under field conditions [Palampur (winter 2017–18 and 2018–19) and Kukumseri (summer 2018)] and in vitro at Palampur [detached leaf method and polyhouse conditions]. Ten lines viz., SP7, SN-1, SN-6-1, SN-7-1, SN-2, SN-5-2, SN-6-2, SN-10, SN-21 and SP-281 showed resistant reaction along with check Palam Sumool while 27 lines were identified as moderately resistant in comparison to susceptible check Azad P-1. Besides, six lines namely, SP-2, SP-5, SP-10, SP-24, SA-4 and SP-12-1 gave moderately susceptible reaction along with checks Pb-89 and Palam Priya. Only, SP-19 was categorized as susceptible. The high yielding lines SP-3, SP-6 and SP-22 showed moderately resistant reaction in both natural and artificial conditions. Validation of resistance using molecular markers revealed that neither the parental genotypes nor the progenies possess the *er1* gene of JI1559. The *er2* linked marker ScOPX-17_1700_ was polymorphic between Palam Sumool and Palam Priya but the marker didn’t show polymorphism between *er2* harboring line (JI2480). These results suggested that the lines showing resistance under field conditions may have some other genes or alleles for resistance and further confirmation is needed by developing mapping populations with specific gene or gene combinations.

## Introduction

Garden pea (*Pisum sativum* L.) is an important nitrogen-fixing vegetable crop of Fabaceae family and forms a significant component of sustainable cropping systems, helps to preserve soil health and output^1^. It is the oldest model of plant genetics and one of the most important legumes in the world^2^. Garden pea is quite palatable and excellent food for human consumption^3,4^ and provides an exceptionally diverse nutrient profile viz*.,* vitamins, minerals and lysine, a limiting amino acid in cereals^1^. Also, fresh pea pods are good source of folic acid, ß-sitosterol, vitamin C and K^5^. Antibacterial, antidiabetic, antifungal, anti-inflammatory, anti-hypercholesterolemia and anti-cancerous properties further support its dietary benefits^6^. India ranks second both in area and production in the world^7^.

Various biotic and abiotic stresses make the local landraces less profitable to farmers due to the reduction in yield. To overcome the further economic loss in the context of biotic and abiotic stresses, there is a dire need to breed resistant and high yielding varieties. Powdery mildew in garden pea caused by *Erysiphe pisi* DC. is a serious disease that can cause 25–50 per cent yield losses^8^. Genetic resistance is quite effective strategy for disease management as it is more cost-effective and environment friendly. The genetics of powdery mildew resistance (PMR) in pea is relatively well understood with three major reported loci *er1*, *er2* and *Er3*^9–11^. Different modes of inheritance *i.e.* single recessive^12,13^, single dominant^14,15^ and duplicate recessive gene action^16,17^ have been reported for powdery mildew resistance. The recessive ‘er1’ gene is responsible for resistance to majority of the naturally occurring powdery mildew disease^18–21^ and was commonly used in pea breeding for development of PMR cultivars. Later, numerous new alleles have been identified which were derived either from natural or artificial mutagenized population^22,23^. The chromosomal position of PMR genes viz*.*, ‘er2’ and ‘Er3’ are known but cloning has not been reported yet. The DNA markers linked to resistance genes provide an alternative to powdery mildew screening and provide an accurate measure as they are not affected by epistatic interactions. They can be used to confirm the presence of multiple resistance genes thereby, increasing efficiency of selection and reducing time span for the introgression of resistance genes.

High yield, specific pod characteristics (proper filling, long, dark green, sweet) and resistant to pests and diseases are the main criteria opted by the breeders for garden pea improvement. The focus on improvement of these specific traits has led to narrowing its genetic base. The varieties like ‘Azad P-1’, ‘Lincoln’, ‘Arkel’ etc. are still preferred by the growers due to desirable horticultural traits though the varieties have become vulnerable to a plethora of biotic and abiotic stresses particularly powdery mildew^24^ and has led to low/stagnant yield and a major impediment in pea improvement. Various studies were conducted for screening and identification of pea genotypes for PMR^25–27^ but these studies have not undertaken validation using molecular markers. In this perspective, four diverse parents selected on the basis of diverse phenological traits were involved in three inter-varietal crosses to isolate transgressive segregants in the recent years that have resulted in 44 progenies with desirable pod characteristics. The present investigation was, therefore, planned with the objective of screening of newly identified genotypes for powdery mildew resistance both in vivo and in-vitro conditions and validation of resistance using molecular markers.

## Material and methods

### Experimental material

The experimental material comprised of 48 genotypes of garden pea of which 44 were advanced breeding lines isolated from three inter-varietal crosses for high pod yield along with four recommended varieties as standard checks. The details are given in the Table 1.

**Table 1:** Genotypes of experiments and their source.

Sr. no	Genotypes	Code	Pedigree (Progeny derived following pedigree method of selection)	Source
1	DPP-SP-1	SP-1	Palam Sumool × Palam Priya	Department of Vegetable Science & Floriculture, CSKHPKV, Palampur
2	DPP-SP-2	SP-2	Palam Sumool × Palam Priya	-do-
3	DPP-SP-3	SP-3*	Palam Sumool × Palam Priya	-do-
4	DPP-SP-5	SP-5	Palam Sumool × Palam Priya	-do-
5	DPP-SP-6	SP-6*	Palam Sumool × Palam Priya	-do-
6	DPP-SP-7	SP-7	Palam Sumool × Palam Priya	-do-
7	DPP-SP-10	SP-10	Palam Sumool × Palam Priya	-do-
8	DPP-SP-12	SP-12	Palam Sumool × Palam Priya	-do-
9	DPP-SP-14	SP-14	Palam Sumool × Palam Priya	-do-
10	DPP-SP-15	SP-15	Palam Sumool × Palam Priya	-do-
11	DPP-SP-17	SP-17	Palam Sumool × Palam Priya	-do-
12	DPP-SP-18	SP-18	Palam Sumool × Palam Priya	-do-
13	DPP-SP-19	SP-19	Palam Sumool × Palam Priya	-do-
14	DPP-SP-22	SP-22*	Palam Sumool × Palam Priya	-do-
15	DPP-SP-24	SP-24	Palam Sumool × Palam Priya	-do-
16	DPP-SN-1	SN-1	Palam Sumool × Pb-89	-do-
17	DPP-SN-4	SN-4	Palam Sumool × Pb-89	-do-
18	DPP-SN-5-1	SN-5-1	Palam Sumool × Pb-89	-do-
19	DPP-SN-6-1	SN-6-1	Palam Sumool × Pb-89	-do-
20	DPP-SN-7-1	SN-7-1	Palam Sumool × Pb-89	-do-
21	DPP-SN-11-1	SN-11-1	Palam Sumool × Pb-89	-do-
22	DPP-SN-15	SN-15	Palam Sumool × Pb-89	-do-
23	DPP-SN-2	SN-2	Palam Sumool × Pb-89	-do-
24	DPP-SN-5-2	SN-5-2	Palam Sumool × Pb-89	-do-
25	DPP-SN-6-2	SN-6-2	Palam Sumool × Pb-89	-do-
26	DPP-SN-7-2	SN-7-2	Palam Sumool × Pb-89	-do-
27	DPP-SN-8	SN-8	Palam Sumool × Pb-89	-do-
28	DPP-SN-10	SN-10	Palam Sumool × Pb-89	-do-
29	DPP-SN-11-2	SN-11-2	Palam Sumool × Pb-89	-do-
30	DPP-SN-13	SN-13	Palam Sumool × Pb-89	-do-
31	DPP-SN-19	SN-19	Palam Sumool × Pb-89	-do-
32	DPP-SN-21	SN-21	Palam Sumool × Pb-89	-do-
33	DPP-SN-22	SN-22	Palam Sumool × Pb-89	-do-
34	DPP-SA-1	SA-1	Palam Sumool × Azad P-1	-do-
35	DPP-SA-4	SA-4	Palam Sumool × Azad P-1	-do-
36	PSPP-8	PSPP-8	Palam Sumool × Palam Priya	-do-
37	DPP-SP-12-1	SP-12-1	Palam Sumool × Palam Priya	-do-
38	DPP-SP-12-2	SP-12-2	Palam Sumool × Palam Priya	-do-
39	DPP-SP-15-1	SP-15-1	Palam Sumool × Palam Priya	-do-
40	DPP-SP-23-1	SP-23-1	Palam Sumool × Palam Priya	-do-
41	DPP-SP-28-1	SP-28-1	Palam Sumool × Palam Priya	-do-
42	DPP-SN-8-2	SN-8-2	Palam Sumool × Pb-89	-do-
43	DPP-SN-3-1	SN-3-1	Palam Sumool × Pb-89	-do-
44	DPP-SN-9-2	SN-9-2	Palam Sumool × Pb-89	-do-
45	Palam Priya	PP	Check	-do-
46	Palam Sumool	PS	Check	-do-
47	Azad P-1	AP-1	Check	CSA University of Agriculture &Technology, Kanpur
48	Punjab-89	Pb-89	Check	Punjab Agriculture University, Ludhiana

*High yielding lines.

### Site of experiment

The experimental material was evaluated at two diverse locations under field conditions at Palampur for two years during winter season (2017–18 and 2018–19) and Kukumseri during summer season of 2018 at 2nd picking and final harvest stage to identify the resistant breeding lines of garden pea against powdery mildew.

### Field screening

Observations on powdery mildew disease severity under field conditions were recorded at peak harvest stage (second/third picking) and also at seed maturity stage macroscopically by following methodology suggested by Banyal and Tyagi^28^. Five infection types were recorded as 0, 1, 2, 3 and 4 scale of Mains and Deitz (Table 2)^29^.

**Table 2:** Infection types and scoring of powdery mildew disease incidence.

Infection type	Reaction	Description
0	Highly resistant (HR)	No mycelium growth
1	Resistant (R)	Sparse mycelium growth with very little sporulation
2	Moderately resistant (MR)	Slight growth of mycelium is evident macroscopically. Microscopically slight to moderate growth of mycelium with conidiophores of the fungus
3	Moderately susceptible (MS)	Moderate growth of mycelium is evident macroscopically. Microscopically moderate development of mycelium with moderate to heavy sporulation is seen
4	Susceptible	Abundant growth of mycelium is evident macroscopically. Microscopically abundant development of mycelium with heavy to very heavy sporulation is visible

### In-vitro screening

The genotypes were also evaluated under in-vitro conditions in polyhouse at Palampur by planting 3–5 seeds of each genotype in individual pots. The in-vitro multiplied conidial inoculum of the disease was dusted on the plants with camel hair brush for uniform development of disease infestation to facilitate screening of genotypes for resistance. Simultaneously, all the lines were also screened by using detached leaf method^28^ under laboratory conditions. Detached leaf assay under in-vitro conditions was conducted using three replications of each variety in the petri dishes under artificial conditions against Palampur isolate of *Erysiphe pisi* along with susceptible and resistant check varieties in the Department of Vegetable Science and Floriculture Laboratory at CSKHPKV, Palampur. The leaves along with petiole were detached from 15 to 30 days old seedlings of each accession and floated on tap water in petri dishes. Fifty ppm of benzimidazole was added to enhance the longevity of the detached floating leaves which were inoculated with the isolate. The petri dishes were incubated at room temperature in laboratory. Observations on the disease development were made at 24 h interval up to twelve days. Infection types were recorded based on macroscopic and microscopic density of mycelia and sporulation at 9 days interval.

### Molecular validation of powdery mildew resistance

#### Isolation, purification and quantification of plant genomic DNA

The total genomic DNA was extracted from all the 48 genotypes and *er1* and *er2* harboring lines (JI- 1559 and JI-2480) followed by PCR amplification using *er1* and *er2* linked markers. The details of molecular markers are presented in Table 3. DNA was isolated from young leaf tissue by using CTAB method given by Murray and Thompson^30^. The extracted DNA samples of all genotypes were loaded on 0.8% agarose gel (1gm/100 ml 1X TAE buffer) and run at 90 V for 40 min to determine the quality and quantity of DNA.

**Table 3:** Detail of markers used for validation.

Sr. no	Name of marker	Sequence	Reference
1	ScOPX-17_1400_	F(5′-GGACCAAGCTCGGATCTTTC-3′)	Katoch et al.^31^
		R(5′-GACACGGACCCAATGACATC-3′)	
2	ScOPE-16_1600_	F(5′-GGTGACTGTGGAATGACAAA-3′)	Tiwari et al.^32^
		R(5′-GGTGACTGTGACAATTCCAG-3′)	
3	AD-60	F(5′- CTGAAGCACTTTTGACAACTAC -3′)	Loridon et al.^33^
		R(5′- ATCATATAGCGACGAATACACC -3′)	

#### Genomic DNA amplification in polymerase chain reaction (PCR)

DNA amplification was carried out in a 12.5 µl reaction volume containing 20 ng template DNA, 0.2 mM of each dNTP, 0.2 µM of each primer, 1.5 mM MgCl_2,_ 1X PCR buffer (10 mM Tris–HCl, 50 mM KCl, pH 8.3) and 1U *Taq* polymerase. PCR amplification for ScOPX17_1400_ and ScOPE16_1600_ carried out in a thermocycler using initial denaturation at 94 °C for 5 min followed by 39 cycles at 94 °C for 30 s, 55 °C for 30 s, 72 °C for 1 min and a final extension at 72 °C for 5 min followed by rapid cooling at 4 °C. The same PCR conditions were used for AD60 except that extension step of the reiterative PCR cycles was carried out for 30 s. The details of markers used for validation is given in Table 3^31–33^.

#### Analysis of PCR product

10 µl of each PCR product was mixed with 3 µl of 6X gel loading dye (0.25% bromophenol blue and 40% sucrose) and electrophoresis was carried out using 4% agarose gel prepared in 1X TAE buffer and ethidium bromide (0.5 µg/ml). The gels were run at a constant voltage of 120 V for 1.5 h. The gel was visualised by using Gel Documentation system (Labnet, ENDURO™ GDS, Aplegen). Presence of appropriate size product of the markers linked to powdery mildew resistant genes was recorded for 48 genotypes.

## Results

### Field screening

The analysis of variance revealed significant differences among 48 genotypes over environments for powdery mildew disease reaction (Table 4). The powdery mildew disease reaction at Palampur during winter season of 2017–18 and 2018–19 revealed that majority of the breeding lines showed resistant to moderately resistant reaction except SP-2 and SA-4 (Table 5; Supplementary Table-1). The SA-4 found to had moderately susceptible reaction during 2nd year. The check variety ‘Palam Sumool’ showed resistant reaction while ‘Pb-89’ was categorized as moderately resistant. Conversely, Palam Priya and Azad-P1 revealed moderately susceptible and susceptible disease reaction, respectively during both the years.

**Table 4:** Analysis of variance for powdery mildew disease incidence over environments.

Source	df	Mean sum of squares					
		Palampur (1st year)	Kukumseri (1st Picking)	Kukumseri (2nd Picking)	Palampur (2nd year)	Poly house conditions	Detached leaf assay
Replications	2	0.05	0.02	0.06	0.11	0.00	0
Treatments	47	1.33	1.46	1.61	1.41	1.76	1.72
Error	94	0.06	0.04	0.04	0.10	0.00	0

**Table 5:** Screening of the genotypes for powdery mildew incidence under field conditions.

Genotypes	Palampur 1st year	Kukumseri (2nd picking)	Kukumseri (last picking)	Palampur 2nd year	Overall	
					Infection type	Reaction type
SP-1	2	2	2	2	2	MR
SP-2	2	1	3	3	3	MS
SP-3	2	1	2	2	2	MR
SP-5	2	1	2	2	2	MR
SP-6	2	2	2	2	2	MR
SP-7	1	1	1	1	1	R
SP-10	2	2	3	2	3	MS
SP-12	2	1	2	2	2	MR
SP-14	2	1	2	2	2	MR
SP-15	2	1	2	2	2	MR
SP-17	2	1	2	2	2	MR
SP-18	1	1	1	1	1	R
SP-19	2	2	3	2	3	MS
SP-22	2	1	2	2	2	MR
SP-24	2	2	3	2	3	MS
SN-1	1	1	1	1	1	R
SN-4	2	1	2	2	2	MR
SN-5-1	1	1	2	1	2	MR
SN-6-1	1	0	1	1	1	R
SN-7-1	1	1	1	1	1	R
SN-11-1	1	2	2	2	2	MR
SN-15	1	1	2	1	2	MR
SN-2	1	0	1	1	1	R
SN-5-2	1	1	1	1	1	R
SN-6-2	1	0	1	1	1	R
SN-7-2	1	1	1	1	1	R
SN-8	1	1	2	2	2	MR
SN-10	1	0	1	1	1	R
SN-11-2	1	1	1	1	1	R
SN-13	1	1	2	1	2	MR
SN-19	1	1	2	2	2	MR
SN-21	1	0	1	1	1	R
SN-22	1	1	1	1	1	R
SA-1	2	1	2	2	2	MR
SA-4	2	2	3	3	3	MS
PSPP-8	1	1	1	1	1	R
SP-12-1	2	1	2	2	2	MR
SP-12-2	2	2	2	2	2	MR
SP-15-1	1	1	1	1	1	R
SP-23-1	1	1	1	1	1	R
SP-28-1	1	1	1	1	1	R
SN-8-2	2	1	2	2	2	MR
SN-3-1	1	1	2	1	2	MR
SN-9-2	2	1	2	2	2	MR
Palam Priya	3	2	3	3	3	MS
Azad-P1	4	4	4	4	4	S
Palam Sumool	1	1	1	1	1	R
Pb-89	2	2	3	2	3	MS
CD (*P* ≤ 0.05)	0.06	0.15	0.16	0.24		

where *R* Resistant; *MR* Moderately resistant; *MS* Moderately susceptible; *S* Susceptible.

**Figure 1:**
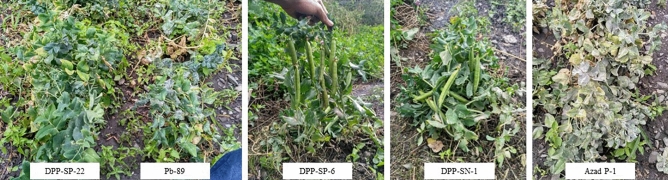
Comparative reaction of different genotypes to powdery mildew disease at Kukumseri.

At Kukumseri during summer 2018, the disease was scored at two harvest stages *i.e.* during second picking and at crop maturity/last picking (Table 5). Azad-P1 witnessed susceptible reaction at early stages (Fig. 1) while 5 newly developed breeding lines were scored as 0 (HR) while 31 and 8 genotypes were rated as 1 (R) and 2 (MR) infection types, respectively at the same growth stage. Of the 36 lines with resistant reaction (Infection types-0 and 1), 17 lines retained infection type 1 till maturity, 19 lines rated as moderately resistant whereas, 5 lines viz., SP-2, SP-10, SP-19, SP-24 and SA-4 recorded moderately susceptible reaction. Checks Pb-89 and Palam Priya were categorized as moderately susceptible with infection type 3 whereas, Palam Sumool showed resistant reaction.

The reaction type of 48 pea lines over the environments (Table 5) exhibited differential response to powdery mildew under field conditions. Among these, 40 lines were resistant (0, 1 and 2 infection types) and 8 were susceptible (3 and 4 infection types). Majority of the newly developed breeding lines showed resistant reaction except SP-2, SP-7, SP-19, SP-24 and SA-4 while Palam Sumool was the only variety with resistant reaction amongst the four checks. The high yielding lines namely, SP-3, SP-6 and SP-22 were moderately resistant as they recorded similar disease reaction (infection type 2) at both the locations Kukumseri and Palampur and therefore, were categorized as moderately resistant.

### In-vitro screening

**Figure 2:**
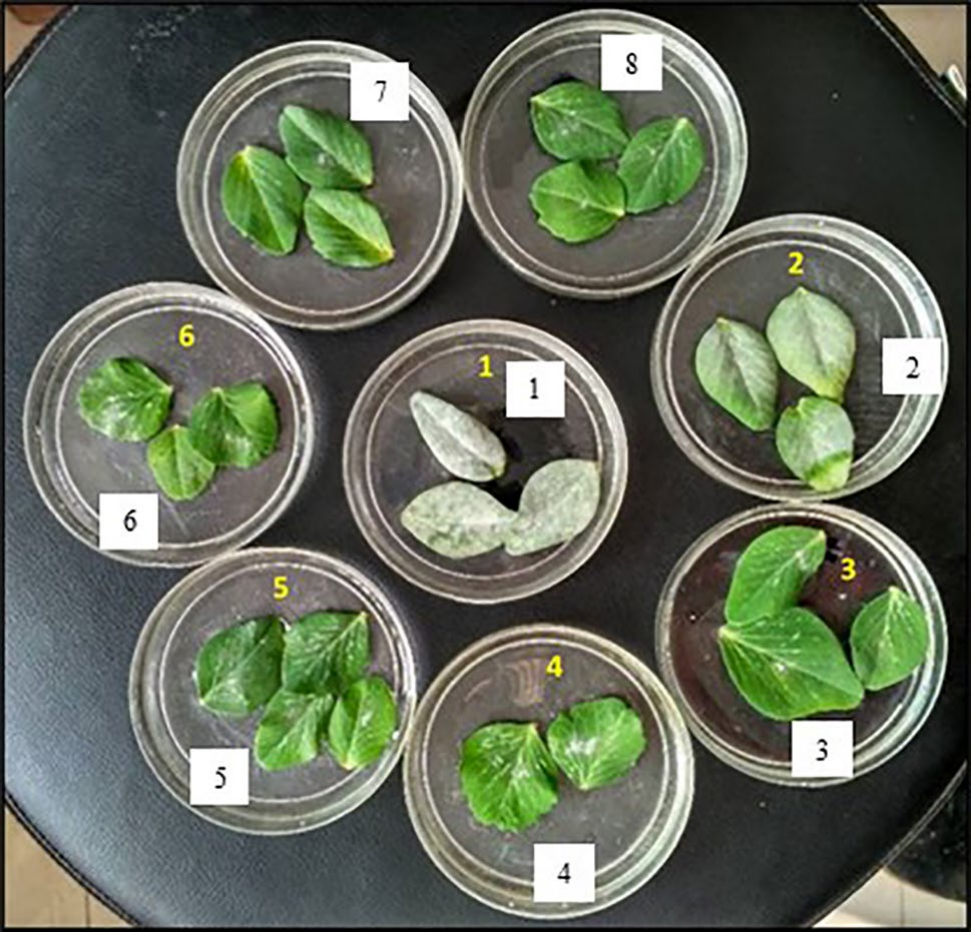
Detached leaf assay with disease reaction of identified desirable genotypes: 1: Lincoln (S), 2: Azad-P1 (S), 3: Pb-89 (MS), 4: SP-6 (MR), 5: SP-22 (MR), 6: SP-3 (MR), 7: Palam Sumool (R), 8: SP-7 (R) where S is susceptible, MS is moderately susceptible , MR moderately resistant, R is resistant.

All these genotypes were also screened in-vitro for powdery mildew disease reaction following detached leaf method and under naturally ventilated polyhouse conditions by raising them in pots. It was observed that out of the 48 lines evaluated under polyhouse conditions, 14, 26, 6 and 2 genotypes exhibited infection type 1 (R), 2 (MR), 3 (MS) and 4 (S), respectively (Table 6; Supplementary Table 2). Broadly, 40 genotypes were classified as resistant (infection types 1 and 2) and 8 as susceptible (infection types 3 and 4) under protected environment. Likewise, detached leaf method also revealed similar disease reactions for majority of the lines with 38 genotypes categorized as resistant (infection types 1 and 2) and 10 as susceptible (infection types 3 and 4). The Fig. 2 depicts the susceptible reaction of Azad-P1and Lincoln, moderately susceptible reaction of Pb-89, moderately resistant reaction of SP-3, SP-6, SP-22 and resistant reaction of Palam Sumool and SP-7.

**Table 6:** Screening of the genotypes for powdery mildew incidence under in-vitro conditions.

Genotypes	Polyhouse		Detached leaf assay		Overall	
	Infection type	Disease reaction	Infection type	Disease reaction	Infection type	Disease reaction
SP-1	2	MR	2	MR	2	MR
SP-2	3	MS	3	MS	3	MS
SP-3	2	MR	2	MR	2	MR
SP-5	2	MR	3	MS	3	MS
SP-6	2	MR	2	MR	2	MR
SP-7	1	R	1	R	1	R
SP-10	2	MR	3	MS	3	MS
SP-12	2	MR	2	MR	2	MR
SP-14	2	MR	2	MR	2	MR
SP-15	2	MR	2	MR	2	MR
SP-17	2	MR	2	MR	2	MR
SP-18	2	MR	2	MR	2	MR
SP-19	4	S	4	S	4	S
SP-22	2	MR	2	MR	2	MR
SP-24	3	MS	3	MS	3	MS
SN-1	1	R	1	R	1	R
SN-4	2	MR	2	MR	2	MR
SN-5-1	2	MR	2	MR	2	MR
SN-6-1	1	R	1	R	1	R
SN-7-1	1	R	1	R	1	R
SN-11-1	2	MR	2	MR	2	MR
SN-15	2	MR	2	MR	2	MR
SN-2	1	R	1	R	1	R
SN-5-2	1	R	1	R	1	R
SN-6-2	1	R	1	R	1	R
SN-7-2	1	R	2	MR	2	MR
SN-8	2	MR	2	MR	2	MR
SN-10	1	R	1	R	1	R
SN-11-2	1	R	2	MR	2	MR
SN-13	2	MR	2	MR	2	MR
SN-19	2	MR	2	MR	2	MR
SN-21	1	R	1	R	1	R
SN-22	2	MR	2	MR	2	MR
SA-1	2	MR	2	MR	2	MR
SA-4	3	MS	3	MS	3	MS
PSPP-8	1	R	2	MR	2	MR
SP-12-1	3	MS	3	MS	3	MS
SP-12-2	2	MR	2	MR	2	MR
SP-15-1	2	MR	2	MR	2	MR
SP-23-1	2	MR	2	MR	2	MR
SP-28-1	1	R	1	R	1	R
SN-8-2	2	MR	2	MR	2	MR
SN-3-1	2	MR	2	MR	2	MR
SN-9-2	2	MR	2	MR	2	MR
Palam Priya	3	MS	3	MS	3	MS
Azad-P1	4	S	4	S	4	S
Palam Sumool	1	R	1	R	1	R
Pb-89	3	MS	3	MS	3	MS

where *R* Resistant; *MR* Moderately resistant; *MS* Moderately susceptible; *S* Susceptible.

Overall, 10 lines viz., SP7, SN-1, SN-6-1, SN-7-1, SN-2, SN-5-2, SN-6-2, SN-10, SN-21 and SP-28-1 showed resistant reaction (infection type 1) along with check Palam Sumool while 27 lines were identified as moderately resistant (infection type 2). In contrary, standard check Azad-P1 and line SP-19 were categorized as susceptible whereas, checks Pb-89 and Palam Priya along with 6 breeding lines namely, SP-2, SP-5, SP-10, SP-24, SA-4 and SP-12-1 revealed moderately susceptible reaction. The summary of disease reaction of various genotypes under field and in-vitro conditions is presented in Table 7.

**Table 7:** Classification of the genotypes based on reaction type under field and in-vitro conditions.

Infection type	Reaction type	Genotypes	
		Field conditions	In-vitro
**1**	R	SP-7, SP-18, SN-1, SN-6-1, SN-7-1, SN-2, SN-5-2, SN-6-2, SN-7-2, SN-10, SN-11-2, SN-21, SN-22, PSPP-8, SP-15-1, SP-23-1, SP-28-1, Palam Sumool	SP-7, SN-1, SN-6-1, SN-7-1, SN-2, SN-5-2, SN-6-2, SN-10, SN-21, SP-28-1, Palam Sumool
**2**	MR	SP-1, SP-3, SP-5, SP-6, SP-12, SP-14, SP-15, SP-17, SP-22, SN-4, SN-5-1, SN-11-1, SN-15, SN-8, SN-13, SN-19, SA-1, SP-12-1, SP-12-2, SN-8-2, SN-3-1, SN-9-2	SP-1, SP-3, SP-6, SP-12, SP-14, SP-15, SP-17, SP-18, SP-22, SN-4, SN-5-1, SN-11-1, SN-15, SN-7-2, SN-8, SN-11-2, SN-13, SN-19, SN-22, SA-1, PSPP-8, SP-12-2, SP-15-1, SP-23-1, SN-8-2, SN-3-1, SN-9-2
**3**	MS	SP-2, SP-10, SP-19, SP-24, SA-4, Palam Priya, Pb-89	SP-2, SP-5, SP-10, SP-24, SA-4, SP-12-1, Palam Priya, Pb-89
**4**	S	Azad-P1	SP-19, Azad-P1

Where *R* Resistant; *MR* Moderately resistant; *MS* Moderately susceptible; *S* Susceptible.

### Validation of resistance using molecular markers

**Figure 3:**
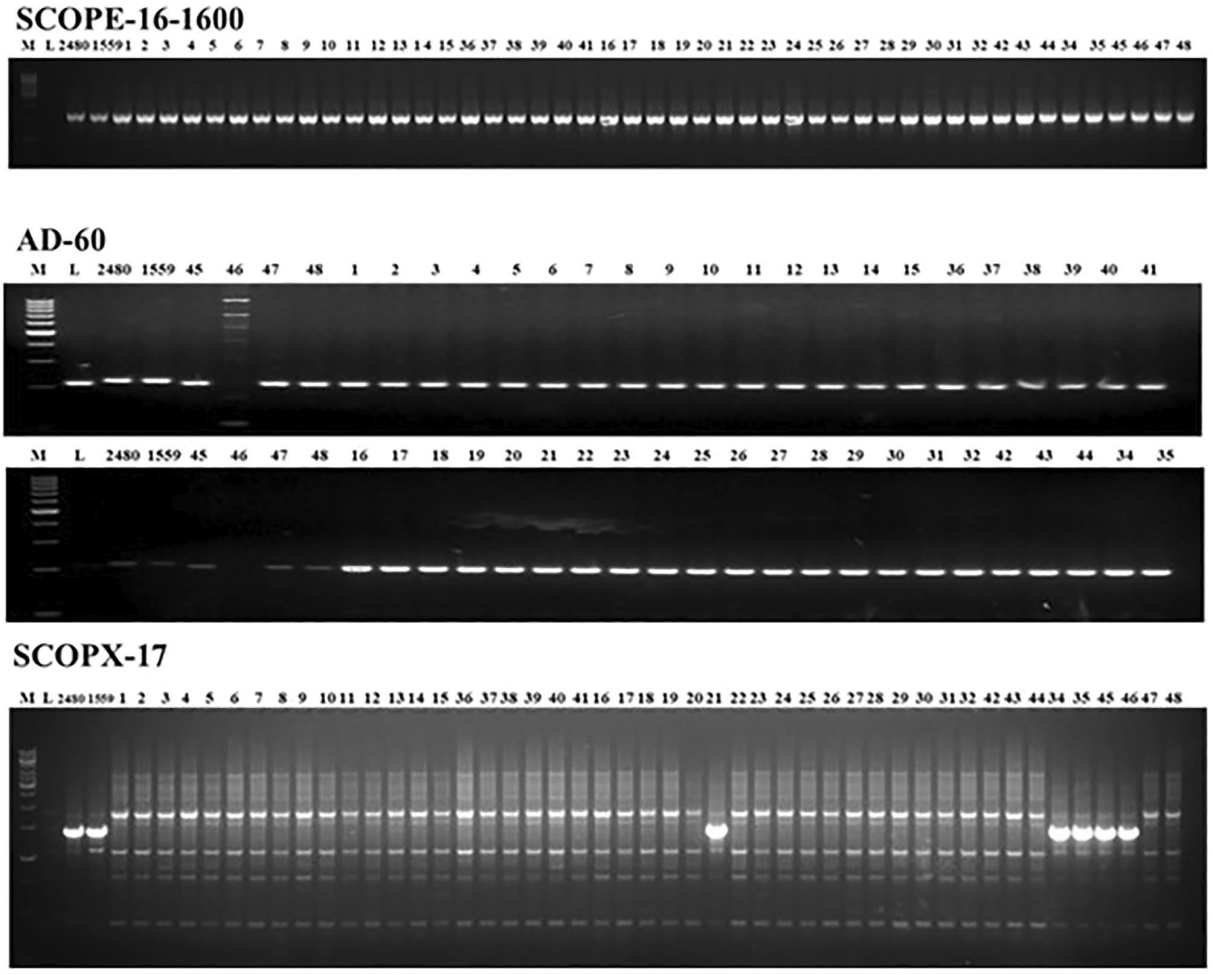
er1 linked markers SCOPE-16-1600 and AD-60 and er2 linked marker SCOPX-17. Legend: M: DNA ladder, L: Lincoln, 2480: er1 harboring line, 1559: er2 harboring line, 1: SP-1, 2: SP-2, 3: SP-3, 4: SP-5, 5: SP-6, 6: SP-7, 7: SP-10, 8: SP-12, 9: SP-14, 10: SP-15, 11: SP-17, 12: SP-18, 13: SP-19, 14: SP-22, 15: SP-24, 16: SN-1, 17: SN-4, 18: 19: 20: 21: SN-11-1, 22: SN-15, 23: SN-2, 24: SN-5-2, 25: SN-6-2, 26: SN-7-2, 27: SN-8, 28: SN-10, 29: SN-11-2, 30: SN-13, 31: SN-19, 32: SN-21, 34: SA-1, 35: SA-4, 36: PSPP-8, 37: SP-12-1, 38: SP-12-2, 39: SP-15-1, 40: SP-23-1, 41: SP-28-1, 42: SN-8-2, 43: SN-3-1, 44: SN-9-2, 45: Palam priya, 46: Azad-P1, 47: Palam sumool, 48: Pb-89.

For validation of powdery mildew resistance, three markers namely, AD60 and ScOPE-16_1600_ linked to *er1* and ScOPX-17_1400_ linked to *er2* were used. The *er1* donor JI1559 and *er2* donor JI 2480 were used for the validation. The amplification products for *er1* and *er2* linked markers are presented in the Fig. 3. The markers linked to ‘Er3’ were not used in this study because the segregation pattern in F_2_ generations of respective cross combinations from which advance breeding lines isolated were observed to be in the ratio of 3 (susceptible): 1 (resistant) indicating the presence of recessive gene in the donor parent (Palam Sumool).

Two markers used for validation of *er1*, the marker ScOPE-16_1600_ was not polymorphic between the *er1* donor JI 1559 and parental genotypes and the amplicons were similar for the progenies as well (Fig. 3) therefore, the marker cannot be used to trace the presence of *er1* in the crosses. The marker AD60 exhibited polymorphism between *er1* donor JI1559 and parental genotypes of crosses Palam Sumool × Palam Priya (Cross-1) and Palam Sumool × Pb-89 (Cross-2). However, all the progeny plants of crosses inherited the non-JI1559 alleles of AD60. Therefore, neither the parental genotypes nor the progenies comprise the *er1* gene of JI1559. This suggests that Palam Sumool and Pb-89 do not contain er1 gene of JI1559.

The *er2* linked marker ScOPX-17_1700_ was polymorphic between Palam Sumool and Palam Priya (Fig. 3) but the marker didn’t show polymorphism between *er2* harboring line (JI2480). Surprisingly, the marker amplified identical amplicons in *er2* harboring line JI2480 and susceptible genotypes Palam Priya and Azad-P1. However, none of the progenies of the cross-1 inherited ScOPX-17_1400_ alleles from either *er2* donor or Palam Priya and the amplicons of progenies match Palam Sumool.

## Discussion

Powdery mildew disease significantly affects the quality and quantity of pea production and is considered as one of the economically important diseases of pea. The use of fungicide is available as an alternative to control the disease but genetic resistance is more favored being more sustainable and eco-friendlier. In the present investigation, four diverse parents selected on the basis of diverse phenological traits were involved in three inter-varietal crosses to isolate transgressive segregants in the recent years that have resulted in 44 progenies with desirable pod characteristics viz., long and dark green pods having high yield and resistance to powdery mildew disease. The powdery mildew resistant progenies were selected in epiphytotic and artificial conditions besides high yield to meet farmer’s preference.

Earlier, many resistant lines have been identified for resistance against powdery mildew using natural and artificial methods^26,34–36^. The validation of resistance in the cultivars can be achieved through in vitro screening by inoculating disease pathogen. However, the recessive nature of the powdery mildew resistance genes and difficulties associated with the handling of obligate pathogens like *Erysiphe pisi,* complicates the selection of resistant phenotypes. During field screening the progression of disease development after first harvest was drastic which may be due to warm days and cool nights favoring disease development. Thompson and Kelly reported that the disease incidence is severe when days are warm and dry and night temperatures are low^37^. Fondevilla and Rubiales also stated that powdery mildew is particularly damaging in late sowings or in late maturing varieties^8^. North-western Indian Himalayan region is natural hot spot for the manifestation of powdery mildew disease^26^. Accordingly at Kukumseri, the disease pressure was higher and disease progressed severely which might be attributed to favourable environmental conditions for disease development and more virulent race of pathogens suggesting it as the hot spot for the disease development (Fig. 1). Based on the high discrimination of the genotypes, Kukumseri was identified as the ideal environment for screening of the genotypes. The variable reaction of genotypes at different locations suggests the difference in virulence of pathogen, variable environmental conditions and variations in the genetic makeup of resistance genes in the accessions^14,26,38^. Due to the variable reaction of the genotypes, there was a need to screen the germplasm under artificial conditions using ample pathogen population.

The genotypes viz., SN-11-2, SP-18, PSPP-8, SP-15-1, SP-23-1, SN-22, SN-7-2 showed resistant reaction in field screening (Table 5) but they were moderately resistant during in-vitro screening (Table 6). Similarly, the disease reaction of SP-12-1 changed from MR to MS and SP-19 from MS to S. This change in the disease reaction of genotypes expressed the actual response of genotypes which showed that the artificial screening methods provide more reliable results as compared to the field screening. This is because in artificial screening favourable conditions are provided to the pathogen for its better perpetuation. The utility of detached leaf assay for screening garden pea for powdery mildew resistance was also mentioned^39^. Similarly, Rana et al.^26^ found 57 accessions out of 701 as resistant to powdery mildew under field conditions but only 14 showed resistance under in vitro conditions. From the results of natural and artificial screening, it can be concluded that high yielding lines viz., SP-3, SP-6 and SP-22 which showed stability for majority of the desirable traits across the environments, also showed moderate resistance both under field and in-vitro conditions (Table 7). Therefore, these lines can be exploited for commercial cultivation even in the hot spot areas.

The DNA markers linked to resistance genes provide an alternative to disease screening of powdery mildew resistance genes and provide an accurate measure as they are not affected by epistatic interactions. They can be used to confirm the presence of multiple resistance genes thereby, increasing efficiency of selection and reducing time span for the introgression of resistance genes. Three monogenic sources of powdery mildew resistance have been identified in pea germplasm, two recessive (*er1 and er2*) and one dominant (*Er3*)^9^.

For validation of powdery mildew resistance, three markers namely, AD60 and ScOPE-16_1600_ linked to *er1* and ScOPX-17_1400_ linked to *er2* were used (Fig. 3). The results suggested that the ScOPX-17_1400_ is not a universally valid marker and cannot be used to confirm the presence of *er2* gene in our parental genotypes and their progenies. The linked markers like ScOPX-17_1400_ and ScOPE-16_1600_ often suffer from the drawback that they show marker haplotypes identical to the resistant parents in many susceptible genotypes thereby limiting their utility in MAS. The main reason attributable to their limitation is that such markers are derived from the genomic regions which have no causative role in the manifestation of resistance and therefore, may be identical in resistant and susceptible genotypes. It is therefore, required that the gene-based markers derived from the functional polymorphisms within the resistance genes should be used to survey the germplasm for the target genes. However, the presence of resistance in many progenies of these crosses suggested that they have inherited some unknown powdery mildew resistance gene different than *er1* from the powdery mildew resistant genotype Palam Sumool. The results also suggest that the lines showing resistance under field conditions may have some other genes or alleles for resistance and further confirmation by developing mapping populations with specific gene or gene combinations is needed^26^ and validation by involving a greater number of markers or developing new markers specific to the gene(s) for powdery mildew resistance.

## Conclusion

Ten lines viz*.*, SP7, SN-1, SN-6-1, SN-7-1, SN-2, SN-5-2, SN-6-2, SN-10, SN-21 and SP-28-1 along with Palam Sumool were identified as resistant along with 27 lines as moderately resistant. High yielding genotypes SP-3, SP-6 and SP-22 were moderately resistant. Molecular markers, used to survey the germplasm revealed that resistance in many of the breeding lines might be inherited from resistant parent Palam Sumool. The molecular markers used in the studies revealed the absence of *er1* in the progenies while *er2* could not be confirmed. Therefore, there is need to involve more number of markers to validate the specific gene providing resistance.

## Supplementary Information

Supplementary Information.

## Data Availability

The datasets generated during and/or analyzed during the current study are presented in the main manuscript and as additional supporting files. Further, any additional information can be obtained from the corresponding author on reasonable request. The advance breeding lines were isolated from diverse intervarietal crosses by the corresponding author and are in accordance with local legislations and comply with institutional/national/international guidelines.

## References

[1] Sharma A, Sekhon BS, Sharma S, Kumar R (2020). Newly isolated inter-varietal garden pea (*Pisum sativum* L.) progenies (F7) under north western Himalayan conditions of India. Exp. Agri..

[2] Kulaeva OA (2017). Pea marker database (PMD)—A new online database combining known pea (*Pisum sativum* L) gene-based markers. PLoS ONE.

[3] Sepehya S, Bhardwaj SK, Dhiman S (2015). Quality attributes of garden pea (*Pisum sativum* L.) as influenced by Integrated Nutrient Management under mid hill conditions. J. Krishi Vigyan.

[4] Gari, A. T. Pea weevil (*Bruchus pisorum* L.) resistance and genetic diversity in field pea (*Pisum sativum* L.). Doctoral Thesis, Swedish University of Agricultural Sciences Alnarp (2015).

[5] Rana C (2021). Stability analysis of garden pea (*Pisum sativum* L.) genotypes under North Western Himalayas using joint regression analysis and GGE biplots. Genet. Resour. Crop Evol..

[6] Rungruangmaitree R, Jiraungkoorskul W (2017). Pea, *Pisum sativum*, and its anticancer activity. Pharmacogn. Rev..

[7] Singh N, Sharma R, Kayastha R (2020). Economic analysis of pea (*Pisum sativum*) in Himachal Pradesh. Econ. Aff..

[8] Fondevilla S, Rubiales D (2012). Powdery mildew control in pea: A review. Agron. Sustain. Dev..

[9] Fondevilla S, Rubiales D, Moreno MT, Torres AM (2008). Identification and validation of RAPD and SCAR markers linked to the gene *Er3* conferring resistance to *Erysiphe pisi* DC. in pea. Mol. Breed..

[10] Srivastava RK, Mishra SK, Singh AK, Mohapatra T (2012). Development of coupling phase SCAR marker linked to the powdery mildew resistance gene er1 in pea (*Pisum sativum* L.). Euphytica.

[11] Pavan S (2013). Identification of a complete set of functional markers for the selection of er1 powdery mildew resistance in *Pisum sativum* L. Mol. Breed..

[12] Harland SC (1948). Inheritance of immunity to mildew in peruvian forms of *Pisum sativum*. Heredity.

[13] Leon DP, Checa OE, Obando PA (2020). Inheritance of resistance of two pea lines to powdery mildew. Agron. J..

[14] Fondevilla S, Torres AM, Moreno MT, Rubiales D (2007). Identification of a new gene for resistance to powdery mildew in Pisum fulvum, a wild relative of pea. Breed. Sci..

[15] Bobkov SV, Selikhova TN (2021). Introgession of powdery mildew resistance into cultural pea from wild accession of P. fulvum. IOP Conf. Ser. Earth Environ. Sci..

[16] Sokhi SS, Jhooty JS, Bains SS (1979). Resistance in pea against powdery mildew. Indian Phytopathol..

[17] Kumar H, Singh RB (1981). Genetic analysis of adult plant resistance to powdery mildew in pea (*Pisum sativum* L.). Euphytica.

[18] Vaid A, Tyagi PD (1997). Genetics of powdery mildew resistance in pea. Euphytica.

[19] Sharma B (2003). The Pisum genus has only one recessive gene for powdery mildew resistance. Pisum Genet..

[20] Janila P, Sharma B (2004). RAPD and SCAR markers for powdery mildew resistance gene er in pea. Plant Breed..

[21] Ek M (2005). Microsatellite markers for powdery mildew resistance in pea (*Pisum sativum* L.). Hereditas.

[22] Humphry M, Reinstädler A, Ivanov S, Bisseling T, Panstruga R (2011). Durable broad-spectrum powdery mildew resistance in pea er1 plants is conferred by natural loss-of-function mutations in PsMLO1. Mol. Plant Pathol..

[23] Pavan S (2011). Pea powdery mildew er1 resistance is associated to loss-of-function mutations at a MLO homologous locus. Theor. Appl. Genet..

[24] Sharma A, Bhardwaj A, Katoch V, Sharma J (2013). Assessment of genetic diversity of garden pea (*Pisum sativum*) as perspective to isolate horticulturally desirable transgressive segregants. Indian J. Agric. Sci..

[25] Davidson JA, Krysinska-Kaczmarek M, Kimber RBE, Ramsey MD (2004). Screening field pea germplasm for resistance to downy mildew (*Peronospora viciae*) and powdery mildew (*Erysiphe pisi*). Australas. Plant Pathol..

[26] Rana JC (2013). Screening of pea germplasm for resistance to powdery mildew. Euphytica.

[27] Ikram A (2020). Screening of resistant germplasm against powdery mildew of pea and its management through nutrients and plant activators. Asian J. Agric. Biol..

[28] Banyal DK, Tyagi PD (1998). Development of powdery mildew of pea in relation to different climatic condition in Himachal Pradesh. Plant Dis. Res..

[29] Mains EB, Dietz SM (1930). Physiologic form of barley, Erysiphe graminis hordei Marchal. Phytopathology.

[30] Murray MG, Thompson WF (1980). Rapid isolation of high molecular weight plant DNA. Nucleic Acids Res..

[31] Katoch V (2010). Molecular mapping of pea powdery mildew resistance gene er2 to pea linkage group III. Mol. Breed..

[32] Tiwari KR, Penner GA, Warkentin TD (1998). Identification of coupling and repulsion phase RAPD markers for powdery mildew resistance gene er-1 in pea. Genome.

[33] Loridon K (2005). Microsatellite marker polymorphism and mapping in pea (*Pisum sativum* L.). Theor. Appl. Genet..

[34] Azmat MA (2010). Single recessive gene controls powdery mildew resistance in pea. Int. J. Veg. Sci..

[35] Rehman A (2014). Estimation of genetic diversity of pea germplasm against powdery mildew (*Erysiphe pisi*) disease and its chemosynthetic management. Pakistan J. Phytopathol..

[36] Banyal DK, Chaudhary J, Singh A (2015). Evaluation of pea (*Pisum sativum*) germplasm for inheritance of resistance to powdery mildew (*Erysiphe pisi*). Indian Phytopathol..

[37] Thompson, H. C. & Kelly, W. C. in *Vegetable Crops*. 5th, New Delhi, Tata McGraw-Hill. (1982).

[38] Banyal DK, Singh A, Tyagi PD (2005). Pathogenic variability in *Erysiphe pisi* causing pea powdery mildew. Himachal J. Agric. Res..

[39] Singh J, Dhall RK, Aujla IS (2015). Characterization of resistance response of garden pea (*Pisum sativum* L.) against powdery mildew (*Erysiphe pisi* DC.) in sub-tropical plains of India. SABRAO J. Breed. Genet..

